# Genome-Wide Identification of WRKY Transcription Factors in Chinese jujube (*Ziziphus jujuba* Mill.) and Their Involvement in Fruit Developing, Ripening, and Abiotic Stress

**DOI:** 10.3390/genes10050360

**Published:** 2019-05-10

**Authors:** Xin Chen, Ruihong Chen, Yanfeng Wang, Cuiyun Wu, Jian Huang

**Affiliations:** 1 Key Comprehensive Laboratory of Forest for Shaanxi Province, College of Forestry, Northwest A&F University, Yangling 712100, China; chenxin0526@126.com; 2 Xinjiang Production & Construction Corps Key Laboratory of Protection and Utilization of Biological Resources in Tarim Basin, College of Plant Science, Tarim University, Alaer 843300, China; wcyby@163.com; 3 Shaanxi Key Laboratory of Chinese Jujube, College of Life Science, Yanan University, Yanan 716000, China; chenruihong328@163.com (R.C.); yadxwyf@yau.edu.cn (Y.W.); 4 Institute of Loess Plateau, Shanxi University, Taiyuan 030006, China

**Keywords:** *Ziziphus jujuba* Mill., WRKY, transcription factors, fruit ripening, drought stress, salt stress

## Abstract

Chinese jujube (*Ziziphus jujuba* Mill.) is an economically important fruit crop in China and mainly cultivated on land with high salinity and drought conditions in northern China. WRKY transcription factors (TFs) are involved in plant development and in responses to multiple abiotic stresses. In this study, we identified 61 and 52 putative ZjWRKY TFs in ‘Junzao’ and ‘Dongzao’ at the genome-wide level. Tissue expression profiling showed that 7 genes were constitutively expressed at high level in all tissues of ‘Junzao’. Transcriptome analysis revealed that 39 *ZjWRKY* genes were expressed during ‘Junzao’ jujube fruit ripening. Among these genes, the transcript abundance of 19 genes were differentially expressed between ‘Junzao’ and ‘Qingjiansuanzao’ fruit. In addition, RT-qPCR analyses revealed that 30, 14, and 18 *ZjWRKY* genes responded to drought, NaCl, and ABA treatments, respectively. Taken together, *ZjWRKY* genes expression dynamics during jujube fruit development, ripening, and their differences between jujube and wild jujube would provide insights into their possible roles regulating fruit ripening. In addition, those *ZjWRKY* genes responded strongly to drought and salt stress, which provide candidate *ZjWRKY* genes for facilitating tolerance breeding.

## 1. Introduction

Chinese jujube (*Ziziphus jujuba* Mill.) is a dominant fruit crops in China that is mainly cultivated in the middle and lower reaches of the Yellow River, a semi-arid region. Since the beginning of the 21st century, the center of jujube cultivation has shifted to arid regions in Northwest China, especially the Xinjiang Autonomous Region [1]. In this region, the jujube cultivation area covered 473,000 ha, accounting for 30% of the total jujube cultivation area in China in 2013, and the corresponding yield was 2.22 million tons, accounting for 51% of the total production of dried jujube in China [2]. In the Xinjiang jujube cultivation area, there is a long sunshine duration (>1200 h), a large temperature difference between day and night (>12 °C), and low rainfall (0.2–9.38 mm) during jujube fruit ripening, all of which might contribute to the jujube fruit quality [3]. For example, the sugar content (73.2%) of ‘Huizao’ jujube produced from Ruoqiang county (N 39.02°, E 88.16°) in the Xinjiang region is significantly higher than the levels seen (59.5%) at their original sites (N 34.54°, E 113.86°) [4]. However, jujube trees are also constantly exposed to extremely adverse conditions in this region, such as soil salinity, drought, and very high or low temperatures. Thus, the jujube tree is likely to have evolved a series of adaptation strategies to cope with such unfavorable conditions [5].

Most abiotic stress, such as high salinity and drought, disrupt the osmotic pressure in plants [6]. In addition, high salt concentrations can lead to ionic toxicity and secondary stress. The Ca^2+^ secondary signal caused by stress could activate related transcription factors (TFs) through the abscisic acid-dependent (ABA-dependent) or mitogen-activated protein kinase pathways [7]. Thereafter, TFs can activate the transcription of specific genes to regulate the physiological and biochemical responses to stress. Thus, TFs play an essential role in the complex regulatory networks of plants. As one of the largest families of TFs in plants, WRKY TFs are involved in regulating plant tolerance to biotic and abiotic stresses, and in plant development [8]. Almost all WRKY proteins contain one or two conserved domains of approximately 60 amino acids containing a conserved heptapeptide WRKYGQK followed by a C_2_H_2_ or C_2_HC zinc finger structure [9]. The WRKY proteins activate or inhibit the expression of target genes by recognizing and binding to a W-box (C/TTGACT/C) in the promoter region of target genes. WRKY TFs are usually divided into three groups, depending on the number of WRKY domains and the type of zinc finger structure. In some studies, WRKY TFs with incomplete zinc finger structures have been assigned to group IV [10].

Several studies have confirmed the function of WRKY TFs regulating plant responses to abiotic stresses. Several *WRKY* genes, such as *AtWRKY33*, *46*, *57* enhance *Arabidopsis thaliana* tolerance to drought/salinity by mediating ABA signal transduction [11,12,13]. In recent years, WRKY TFs identification at the genome-wide level have been facilitated by the greater availability of the genome sequences of fruit crops, such as *Malus domestica* [14], *Citrus sinensis* [15], and *Vitis vinifera* [10]. The functions of some WRKY genes have also been further verified in some species. In *Fortunella crassifolia*, *FcWRKY40* enhances salt tolerance by regulating ion homeostasis and proline synthesis dependent on the ABA signal transduction pathway [16]. *VvWRKY30* positively regulates drought response by modulating proline and soluble sugar metabolism, as well as activating reactive oxygen scavenging systems [17]. Considering the vital role of WRKY TFs response to multiple stresses, it would be valuable to characterize the WRKY TFs in the jujube genome.

In addition to responding to abiotic stresses, WRKY TFs are also involved in a variety of biological processes such as leaf senescence [18], trichome, seed and pollen development [19,20], and secondary metabolite biosynthesis [21]. Recently, some studies have revealed that WRKY TFs may be involved in fruit development and ripening [22,23]. Jujube was domesticated from its wild ancestor (*Z. jujuba* Mill. var. spinosa Hu.), which resulted in enlarged fruit sizes and changes in fruit taste such as increased sweetness/acidity. In our previous report, we have elucidated the difference in fruit taste between the wild jujube and jujube fruit through transcriptomic analysis and genomic selection [24]. However, there were still lack of detail information about TFs involved in jujube fruit development and ripening. Therefore, it is valuable to study the expression pattern of *WRKY* genes, one of the largest plant TF families, and their differential expression between jujube and wild jujube during fruit ripening, and hope to reveal the potential role of *WRKY* genes involved in fruit ripening.

In this study, we identified and compared WRKY TFs in two genome-sequenced jujube cultivars, i.e., ‘Junzao’ and ‘Dongzao’, at the genome-wide level, and characterized their chromosomal location and phylogenetic relationships. ‘Junzao’ is a dominant dry-eating jujube cultivar with large size and high sugar content, while ‘Dongzao’ is the most dominant fresh-eating cultivar with crisp flesh and good tastes in China. We analyzed the expression profiles of WRKY TFs in different tissues of ‘Junzao’. To elucidate their potential role in regulating fruit development and ripening, we compared the transcription patterns of cultivated and wild jujube during fruit ripening. Finally, we performed pot and hydronic experiments and studied the responses of different *ZjWRKY* genes to drought stress, salt stress, and ABA treatment using RT-qPCR. The results would help us understand the molecular mechanisms of jujube adaptation to harsh conditions and provide gene resources for jujube molecular breeding to improve stress tolerance and fruit quality.

## 2. Materials and Methods

### 2.1. Identification of the WRKY Family Members in Chinese Jujube and Their Chromosomal Locations

The protein-coding sequences and their corresponding chromosomal locations in the jujube genome were downloaded from our previously released data of ‘Junzao’ jujube genome (https://datadryad.org/resource/doi:10.5061/dryad.83fr7). The Hidden Markov Model profiles for the WRKY DNA-binding domain (PF03106) were retrieved from the Pfam data base (http://pfam.xfam.org/) [25] and used to identify jujube *WRKY* genes (E-Value < 0.01) with HMMER 3.0 (http://hmmer.janelia.org/) [26]. Pfam domains and NCBI CDD (http://www.ncbi.nlm.nih.gov/cdd/) were used to validate all the candidate WRKY TFs. We also identified *WRKY* gene family in ‘Dongzao’ jujube genome (https://www.ncbi.nlm.nih.gov/genome/?term=jujube). We compared amino acids composition of WRKY TFs between ‘Junzao’ and ‘Dongzao’ using BlastP.

### 2.2. Sequence Alignment of WRKY Family Members and Construction of the Phylogenetic Tree

Conserved domain amino acid (aa) sequences of all predicted ZjWRKY were multi-aligned using ClustalX 2.0.12 [27]. Another multi-alignment, including aa sequences of jujube and those from *A. thaliana* and *Solanum lycopersicum*, was performed using ClustalW and MEGA 7.0 with the neighbor-joining and maximum likelihood method (bootstrap = 1000) [28]. Based on the results of multiple sequence alignment and the classification of *A. thaliana* WRKY TFs, all predicted *ZjWRKY* genes were assigned to groups and subgroups.

### 2.3. Structural and Analysis of WRKY Genes

The online ExPASy proteomics server (https://web.expasy.org/protparam/) was used to predict protein molecular weights (MWs) and isoelectric points (pIs) of the putative WRKY proteins. Motifs in all the predicted ZjWRKY protein sequences were identified using the Multiple Em for Motif Elicitation (MEME) 5.0.1 online program (http://meme-suite.org/tools/meme) [29]. The parameters for MEME were as follows: number of repetitions, any; maximum number of motifs, 10; and the optimum width of each motif, between 20 and 50 residues. To evaluate the level of conservation of the WRKY structural domains and zinc finger motifs of each group, sequence logos were produced using Jalview 2.10.4b1.

Both gene sequence and coding sequence (CDS) of each predicted *ZjWRKY* gene from the *Z. jujuba* genome were downloaded, and the intron distribution pattern and splicing phase were analyzed using the gene structure display server (GSDS) (http://gsds.cbi.pku.edu.cn/) [30].

### 2.4. Analysis of Cis-Acting Regulatory Elements in the Promoter Regions of ZjWRKY Genes

We extracted the region of 2000 bp upstream of the transcriptional start point of *ZjWRKY* genes from ‘Junzao’ and ‘Dongzao’ jujube genome [22], and identified *cis*-acting regulatory elements in these regions using PlantCARE [31].

### 2.5. Tissue-Specific Expression Analysis of ZjWRKY Genes by Reverse Transcription Polymerase Chain Reaction (RT-PCR)

We performed RT-PCR analysis to study the *ZjWRKY* genes expression in different tissues, including roots, stems, flowers, and leaves of ‘Junzao’. We collected at least 30 g leaves, stems and fine roots, 30 young fruits, 10 ripe fruits, and 3 g flower samples from one tree per replicate, respectively. Three biological replicates for each type of tissues were collected totally. These samples were harvested on June 16, 2017, from three 10-year-old ‘Junzao’ jujube trees growing in the Jujube Experimental Station of Northwest A&F University (Qingjian, Shaanxi, China; N 37.13°, E 110.09°). Over 50% flowers were fully blooming on June 16, 2017 in the experimental station and this time was defined as 0 days after flowering (DAF), young fruits were collected at 15 DAF (1 July 2017) and ripe fruits were collected at 110 DAF (4 October 2017). In addition, we dissected flowers into 4 parts, included sepals, disks, pistils, and stamen. All prepared samples were frozen in liquid nitrogen and then transferred to a −80 °C freezer for storage.

Total RNA was extracted using a Plant RNA Extraction Kit (Takara, Dalian, China) and DNA was removed using DNase I. The RNA was quantified using a NanoDrop 2000 UV-vis spectrophotometer (Thermo Fisher Scientific, USA), and its integrity was confirmed using 1% agarose gel electrophoresis. cDNA was synthesized by the PrimeScript™ RT reagent Kit (Takara, Dalian, China). Primer sets for 61 ZjWRKY genes identified from ‘Junzao’ jujube were designed by NCBI Primer-BLAST (https://www.ncbi.nlm.nih.gov/tools/primer-blast/). The parameters for Primer-BLAST were as follows: PCR product size, 80~180bp; melting temperatures (Tm), 57~60; the forward/reverse primer range was specified to span one intron; forward/reverse primer length: 19~22 bp, GC%: 50~55, self complementarity: 0~6, self 3′ complementarity: 0~3. ZjUBQ (GenBank Accession: EU916200.1; Forward primer: 5′-TGGATGATTCTGGCAAAG-3′; Reverse primer: 5′-GTAATGGCGGTCAAAGTG-3′) was selected as reference for RT-PCR analysis [32]. RT-PCR was performed using 2×GoldStar BesterMix (CWBIO, Beijing, China) on an ABI 2720 Thermal Cycler (ABI, Marsiling, Singapore). PCR products were detected by 1.2% agarose electrophoresis. Three technical replicates were performed for each sample. The band intensity was detected using Image J [33].

### 2.6. Transcriptome Analysis of ZjWRKY Genes During Jujube and Wild Jujube Fruit Development and Ripening

Wild jujube is the wild ancestors of jujube, which have small fruit size and acidity taste in contrast cultivated jujubes with large fruit size and sweet taste [24]. To identify the WRKY TFs that may be associated with jujube fruit size enlargement or sugar accumulation, we compared their expression level of WRKY TFs between wild and cultivated jujube fruits based on transcriptomic analysis. We harvested cultivated jujube (‘Junzao’) and wild jujube (‘Qingjiansuanzao’) fruits at five stages of fruit ripening: young (15 DAF), enlarging (40 DAF), white mature (65 DAF), beginning red (90 DAF), and fully red (110 DAF) from 10-year-old trees, respectively, which were grown in Jujube Experimental Station of Northwest A&F University under the same environment and management. Three biological replicates were harvested, with at least 10 fruits (30 fruits at the first stage) collected from one tree per replicate. RNA-seq were performed by sequencing 250~300 bp paired-end libraries on Illumina Novaseq platforms. Sequencing were performed at the Institute of Novogene (Beijing, China). Gene expression was analyzed refer to the released ‘Junzao’ genome. We defined genes with reads per kilobase per million mapped reads (RPKM) ≥ 1 as being expressed.

### 2.7. Salt Stress, Drought Stress, and ABA Treatment Experiments

#### 2.7.1. Seedling Preparation

Wild jujubes are genetically close to cultivated jujube and usually used as the rootstock of jujube cultivars [24]. In this study, we evaluated the expression of *ZjWRKY* genes of wild jujube responding to drought/salt resistance. Wild jujube seeds were collected from the State Forestry Administration Forest Tree Germplasm (Xingtai, China). Seeds were rinsed with tap water for 24 h and sowed in sterilized soil (peat soil: sand = 3:1). All seedlings were grown under natural light in the greenhouse of Northwest A&F University and the temperature was 25 ± 3 °C. Seven-week-old seedlings with similar height and biomass were selected for subsequent experiments.

#### 2.7.2. Drought Stress Treatment

In first, 45 selected seedlings were transplanted from the substrates to pots. At first, the soil moisture in the pots was kept at 60% for 7 days, then stop watering and soil moisture naturally decreased. When the soil moisture reached 60%, 40%, 20%, and 10%, and kept for 3 days at each level, 9 seedlings were harvested, respectively. The remained 9 seedlings were re-watered. When the soil moisture was raised to 60% and kept for 3 days (REW60%), the seedlings were harvested. In this experiment, the soil water content of each pot was monitored and adjusted by a weighing method [34].

#### 2.7.3. NaCl Treatment

In total, 45 selected seedlings were transplanted from the substrates to PVC boxes containing 6 L ½ Hoagland solution. Prior to NaCl treatment, seedlings were grown in ½ Hoagland solution 5 days for adaptation to the hydroponic conditions. Then, the solution was replaced with ½ Hoagland nutrient solution containing 200 mM NaCl [35]. When the treatment time reached 0, 3, 12, 24 h, 9 seedlings were harvested, respectively. The remaining 9 seedlings were transplanted to ½ Hoagland nutrient solution without additional NaCl, and harvested after 12 h (RS12h). Ventilation was maintained throughout hydroponic growth.

#### 2.7.4. ABA Treatment

The ABA treatment was similar to the NaCl treatment except that 200 mM NaCl was replaced by 100 μM ABA (Yuanye, Shanghai, China).

### 2.8. RT-qPCR Analyses of ZjWRKY Genes in Response to Abiotic Stresses

Gene transcription levels were quantified by RT-qPCR using TaKaRa TB Green™ Premix Ex Taq ™ II (Dalian, China) on a Bio-Rad CFX Connect system (CA, USA). We made the standard curves using a 10-fold dilution series of the cDNA templates from 7-week old seedling without any treatment. The PCR amplification efficiency of each primer pair was evaluated based on the slope of a standard curve for each gene. In addition, we performed the melting curve analysis to confirm the specificity of the primer pairs. Those primers designed for tissue-expression were firstly subjected to evaluation, and we only accepted the primer sets with the amplification efficiencies ranging between 90% and 110% and melting curve showing single peak. Each 10 μL reaction mixture was composed of: 5 μL TB Green Premix Ex Taq II, 0.8 μL 10 μM primer set, 1 μL cDNA solution, and 3.2 μL ddH_2_O. Cycling conditions were set as 95 °C for 30 s followed by 40 cycles at 95 °C for 5 s and 60 °C for 30 s. Melting curve analysis was carried out under the following cycling conditions: 95 °C for 5 s followed a gradient of 65 °C to 95 °C where each increase of 0.5 °C lasted 5 s. The expression of ZjWRKY genes was defined based on the cycle threshold (Ct), and their relative expression levels were calculated as 2^−ΔΔCt^ after normalization to the expression of *ZjUBQ* as the reference gene [36]. The Venn diagram was drawn using TBtools [37].

## 3. Results

### 3.1. Identification and Chromosomal Locations of ZjWRKY Family in Jujube

**Figure 1:**
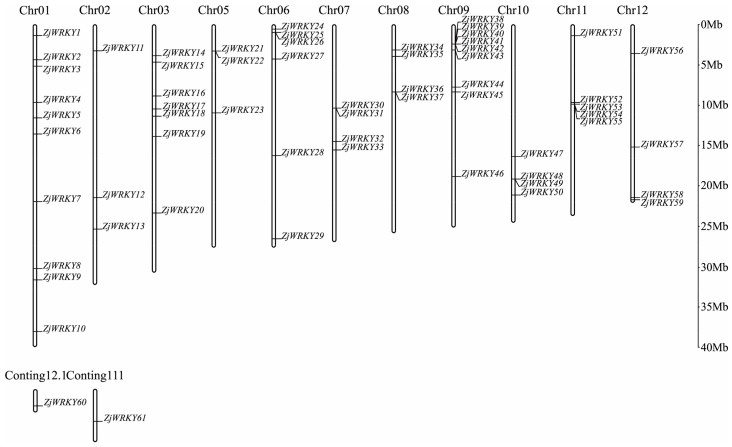
Distribution of *ZjWRKY* genes on pseudo chromosomes of ‘Junzao’. The scale on the right is in million bases (Mb).

In this study, a total of 61 potential *ZjWRKY* genes were identified through genome-wide analysis and designated according to their chromosomal location of ‘Junzao’ jujube genome (Table 1). All 61 predicted genes containing WRKY domains were confirmed. The length of these 61 proteins varied from 158 (*ZjWRKY60*) to 758 (*ZjWRKY4*) amino acids. The predicted MWs ranged from 18 kDa to 82 kDa, and the pIs varied from 5.16 (*ZjWRKY52*) to 9.53 (*ZjWRKY59*). Among these 61 ZjWRKY genes, 59 were located on the 11 jujube chromosomes, and chromosome 4 did not contain any *ZjWRKY* gene. 10 *ZjWRKY* genes were mapped on chromosome 1, which contained the most *ZjWRKY* genes. Chromosomes 2 and 5 each contained only 3 *ZjWRKY* genes (Figure 1).

On the other hand, a total of 52 WRKY TFs were identified from the ‘Dongzao’ jujube genome, which distributed on 11 putative chromosomes. There was no *ZjWRKY* gene on chromosome 7 (Figure S1a). We found 13 members showing the same sequencing composition between the two cultivars, and 33 WRKY TFs member in ‘Dongzao’ have their corresponding members in ‘Junzao’ with high identity (>90%). However, there were still 6 members showing low identity (53–86%) (Table S1).

### 3.2. Phylogenetic Classification of ZjWRKY Genes

**Figure 2:**
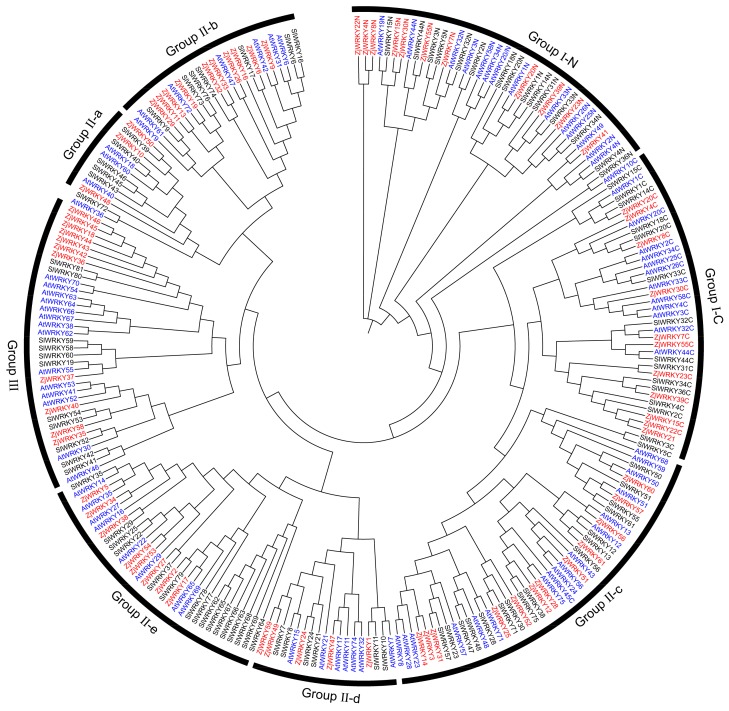
Phylogenetic analysis of the WRKY proteins from *Ziziphus jujuba* ‘Junzao’ jujube, *Arabidopsis thaliana* and *Solanum lycopersicum*. Multiple sequence alignments of WRKY amino acid sequences were performed using ClustalW. The phylogenetic tree was constructed using MEGA7.0 with the neighbor-joining method and 1000 bootstrap replicates (substitution model: Jones–Taylor–Thornton model). Group (I, II, III) and subgroup (IIa~IIe) names were marked outside the circle.

ZjWRKY TFs of ‘Junzao’ jujube were classified into three groups based on the number of WRKY domains and the type of zinc finger motif. Group I contained two WRKY motifs (one in the N-terminal region of the sequence, the other in the C-terminal region) and two C_2_H_2_ zinc finger motifs; 10 ZjWRKYs were assigned to this group. Group II contained a WRKY motif and a C_2_H_2_ zinc finger motif, except for *ZjWRKY53*. This group contained 40 members. Group III included 11 members, and each of them contained a WRKY motif followed with a C_2_HC zinc finger motif, except *ZjWRKY18* (Figure 2 and Table 1). On the other hand, 52 WRKY TFs of ‘Dongzao’ jujube were also divided into 3 groups which were group I with 10 members, group II with 30 members, group III with 12 members. In contrast to ‘Junzao’, WRKY TFs in group II of ‘Dongzao’ was obviously reduced (Table S2 and Figure S1b).

For further classification, WRKY proteins from three different plant species, including 71 from *A. thaliana*, 77 from *S. lycopersicum*, and 61 from *Z. jujuba* ‘Junzao’, were subjected to phylogenetic analysis. Group II was divided into five subgroups (IIa to IIe) according to the zinc finger structure (Figure 2 and Figure S2). Subgroups IIa, IIb, IIc, IId, and IIe contained 3, 10, 14, 5, and 8 members, respectively. In ‘Dongzao’ jujube, subgroups IIa, IIb, IIc, IId, and IIe contained 2, 8, 12, 3, and 5 members, respectively (Table S2). The zinc finger structure from the group IN was C-X_4_-C-X_22_-H-X_1_-H, and IC was C-X_4_-C-X_23_-H-X_1_-H. The members of subgroups IIa, IIb, IId, and IIe had C-X_5_-C-X_23_-H-X_1_-H zinc-finger motifs, except for the ZjWRKY53. Group IIc had C-X_4_-C-X_23_-H-X_1_-H zinc finger structure. Group III had a C-X_7_-C-X_23_-H-X_1_-C zinc finger motif, except for ZjWRKY18, which had no complete zinc-finger structure (Figure S3). We found 4 members (XP_015874714.1, XP_024928884.1, XP_024933427.1, and XP_015902857.1) in group III did not have a complete zinc finger structure in ‘Dongzao’ jujube (Figure S1g).

Although the WRKY domain was highly conserved, there were some mutations in Chinese jujube. The WRKY motifs (WRKYGQK) had one amino acid modification (WRKYGKK) in ZjWRKY57 and ZjWRKY60 that belonging to group IIc, whereas in ZjWRKY45 (group III), the WRKY domain was replaced by WRQY (Figure S3). In ‘Dongzao’ jujube, group IIc (XP_015884439.1 and XP_015870123.1) also contained the aa sequence WRKYGKK. The mutations in the conserved domain of these WRKY genes have been confirmed by sanger sequencing in ‘Junzao’ jujube. In addition, we identified the orthologous genes (*AtWRKY51* and *AtWRKY50*) of *A. thaliana* regarding to *ZjWRKY57* and *ZjWRKY60* by phylogenetic analyses and also found the mutation (WRKYGKK) in these proteins. However, we did not find an orthologous gene of *ZjWRKY45* in *A. thaliana* and *S. lycopersicum*.

### 3.3. Conserved Motifs of the ZjWRKY and Structure of Their Genes

**Figure 3:**
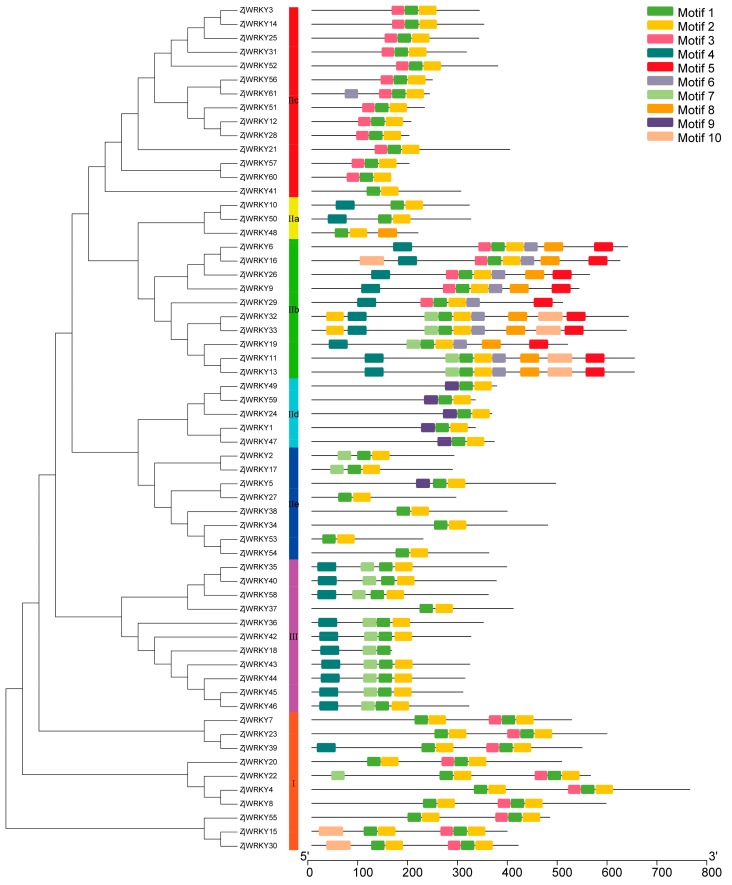
Phylogenetic tree and motif composition of ‘Junzao’ WRKY proteins. The phylogenetic tree was constructed using MEGA7.0 with the neighbor-joining method and 1000 bootstrap replicates (substitution model: Jones–Taylor–Thornton model). Multiple Em for Motif Elicitation (MEME) was used to predict motifs. Ten motifs were identified and were represented by different colors. The position of the motif on the chromosome was labeled.

MEME motif analysis identified 10 motifs in all WRKY members of two Chinese jujube cultivars. Each ZjWRKY had different motif combinations. Motif 1 was annotated as WRKY DNA-binding motif, which is the fundamental characteristic of WRKY TFs (Figure S4) and presented in all ZjWRKY at least one time (Figure 3 and Figure S1c). Motif 2 was zinc finger motif and almost presented in all ZjWRKYs except two members (ZjWRKY18 and XP 015902857.1) belonging to group III. Group I proteins had two WRKY domains, each consisting of the conserved aa sequence WRKYGQK (motif 1) and a zinc finger (motif 2), except XP 015892560.1 of ‘Dongzao’. Motif 5 was unique to group IIb. Motif 6 only presented in group IIb of ‘Dongzao’ jujube while it presented in both group IIb and group IIc (ZjWRKY61) of ‘Junzao’ jujube.

**Figure 4:**
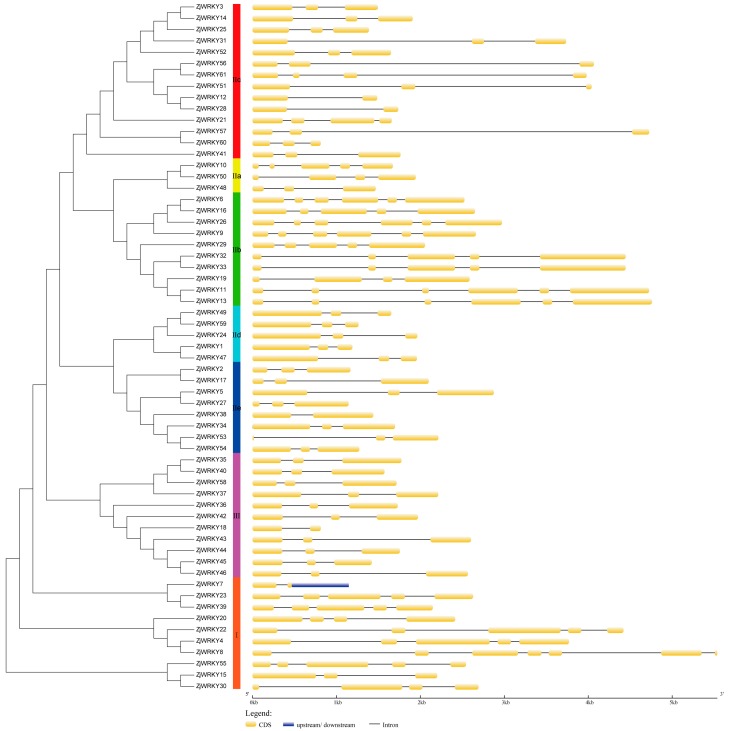
Intron-exon structure of *ZjWRKY* genes of *Ziziphus jujuba* ‘Junzao’. The phylogenetic tree was constructed using MEGA7.0 with the neighbor-joining method and 1000 bootstrap replicates (substitution model: Jones–Taylor–Thornton model). Analysis of *ZjWRKY* genes structure using gene structure display server (GSDS). Yellow blocks indicate the coding sequences (CDS), blue blocks indicate upstream or downstream sequences, and black lines indicate introns.

Gene structure predictions revealed that all *ZjWRKY* genes contained CDS and introns. The number of CDS varied from 2 to 7. *ZjWRKY* genes containing 3 CDS were the most frequent type and accounted for 55.74% (Figure 4). In similar, *ZjWRKY* containing 3 CDS was the most dominant type in ‘Dongzao’ jujube (Figure S1d).

### 3.4. Cis-Acting Regulatory Elements of the ZjWRKY Promoters

**Figure 5:**
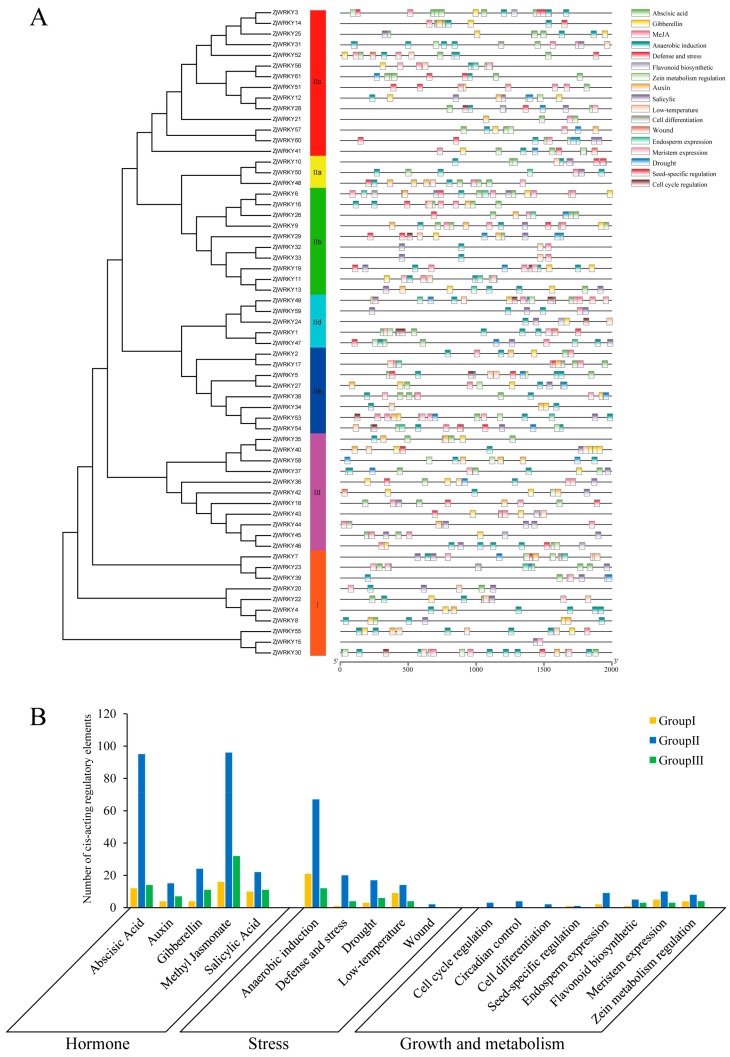
Type and number of *cis*-acting regulatory elements in the promoter region of *WRKY* genes of *Ziziphus jujuba* ‘Junzao’. (**A**) Position of the *cis*-acting elements in the *ZjWRKY* genes promoter region. (**B**) Number of *cis*-acting elements.

These *cis*-acting regulatory elements in the ‘Junzao’ *ZjWRKY* genes promoter regions were mainly classified into four categories: light-responsive elements, hormone-responsive elements, stress-responsive elements, and growth and metabolic-responsive elements (Figure 5a,b). The light-responsive elements were present in the promoter regions of all *ZjWRKY*, and included Box 4, G-box, and GT1-motif. Hormone-responsive elements included response elements to ABA (ABRE), auxin (AuxRR-core, and TGA-box), gibberellin (GARE-motif, P-box, and TATC-box), methyl jasmonate (CGTCA-motif, and TGACG-motif), and salicylic acid (TCA-element), among which the methyl jasmonate (MeJA)-responsive elements were the most dominant, accounting for 37.36% of the hormone-responsive elements. The stress-responsive elements included response elements to anaerobic induction (ARE), defense and stress (TC-rich repeats), drought (MBS), low temperature (LTR) and wound (WUN-motif). Among these, response elements to anaerobic induction were the most abundant, accounting for 55.00% of stress-responsive elements. We found hormone-responsive and stress-responsive elements present in the promoter region of some *ZjWRKY* genes in each of the three groups. Growth and metabolic-responsive elements mainly include: elements associated with Zein-metabolism regulation (O_2_-site), meristem expression (CAT-box), and endosperm expression (GCN4_motif). In addition, we found the W-box element in the promoter regions of the 43 *ZjWRKY* genes (Table S3). Similar to the ‘Junzao’ jujube, the light-responsive elements were observed in the promoter region of all ‘Dongzao’ jujube *WRKY* genes. Unlike the ‘Junzao’ jujube, the ABA-responsive elements were the most dominant among the hormone-responsive elements, and *cis*-acting regulatory element involved in zein metabolism regulation was the most dominant among the growth and metabolic-responsive elements (Figure S1e,f).

### 3.5. Tissue-Specific Expression Profile of ZjWRKY Genes

**Figure 6:**
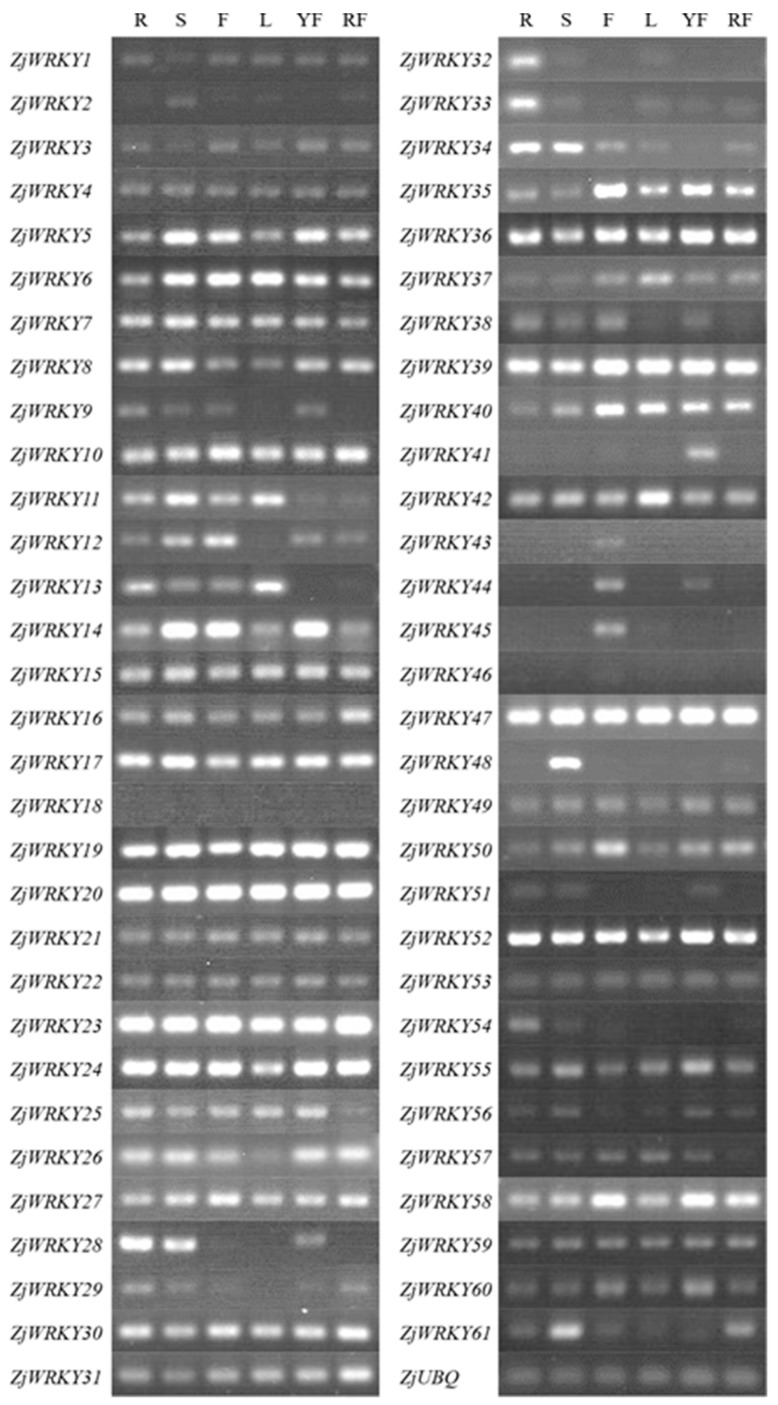
RT-PCR analysis of *ZjWRKY* genes in six types of tissues/organs of ‘Junzao’ jujube. R: root; S: stem; F: flower; L: leaf; YF: young fruit; and RF: ripe fruit.

To investigate whether the predicted ‘Junzao’ *WRKY* genes were actually transcribed, we examined their transcription levels in different tissues using RT-PCR. We designed primer sets for each candidate *ZjWRKY* genes (Table S4). RT-PCR analyses showed that 41 genes were transcribed in roots, stems, flowers, leaves, young fruit and ripe fruit, but their expression levels varied among these tissues (Figure 6). As shown in Figure 6, seven genes (*ZjWRKY19*, *20*, *23*, *24*, *39*, *47*, and *52*) highly expressed in all six tissues. Further analysis of the promoter regions of these 7 genes revealed that ABA-responsive element was the dominant type followed by anaerobic induction and SA-responsive element (Table S3). No bands were detected in all tissues for two genes (*ZjWRKY18*, and *46*). *ZjWRKY46* was activated by the following drought, NaCl and ABA treatments while *ZjWRKY18* was still not detected under such treatments (Figure S5). We identified 1 ABA-responsive element, 1 SA-responsive element and 1 defense and stress-responsive element in the promoter region of *ZjWRKY46* and 2 MeJA-responsive elements, 1 ABA-responsive element and 1 defense and stress-responsive element in the promoter region of *ZjWRKY18* (Table S3). *ZjWRKY32*, and *33* in group IIe were specifically expressed in roots. There were 4 MeJA-responsive elements, 4 anaerobic induction-responsive elements and 2 low temperature-responsive elements in the promoter regions of these 2 genes. *ZjWRKY41*, in group IIc, was specifically expressed in young fruit (15 DAF), and *ZjWRKY43*, and *45*, in group III, were only detected in flowers. We found 1 GA-responsive element was common in the promoter regions of *ZjWRKY41*, *43* and *45* (Table S3).

We further examined the expression patterns of *ZjWRKY43* and *45* in floral organs, including sepals, disks, pistils, and stamen, by RT-PCR. *ZjWRKY43* was expressed in pistils and disks, and *ZjWRKY45* was expressed in pistils, sepals and disks. Neither were expressed in the stamen (Figure S6). In addition, the transcription levels of 15 *ZjWRKY* genes (*ZjWRKY9*, *12*, *14*, *25*, *28*, *29*, *34*, *38*, *41*, *44*, *51*, *55*, *57*, *60*, and *61*) changed between young and ripe fruits (Table S5).

### 3.6. Transcriptional Dynamics of ZjWRKY Genes During Fruit Development and Ripening

**Figure 7:**
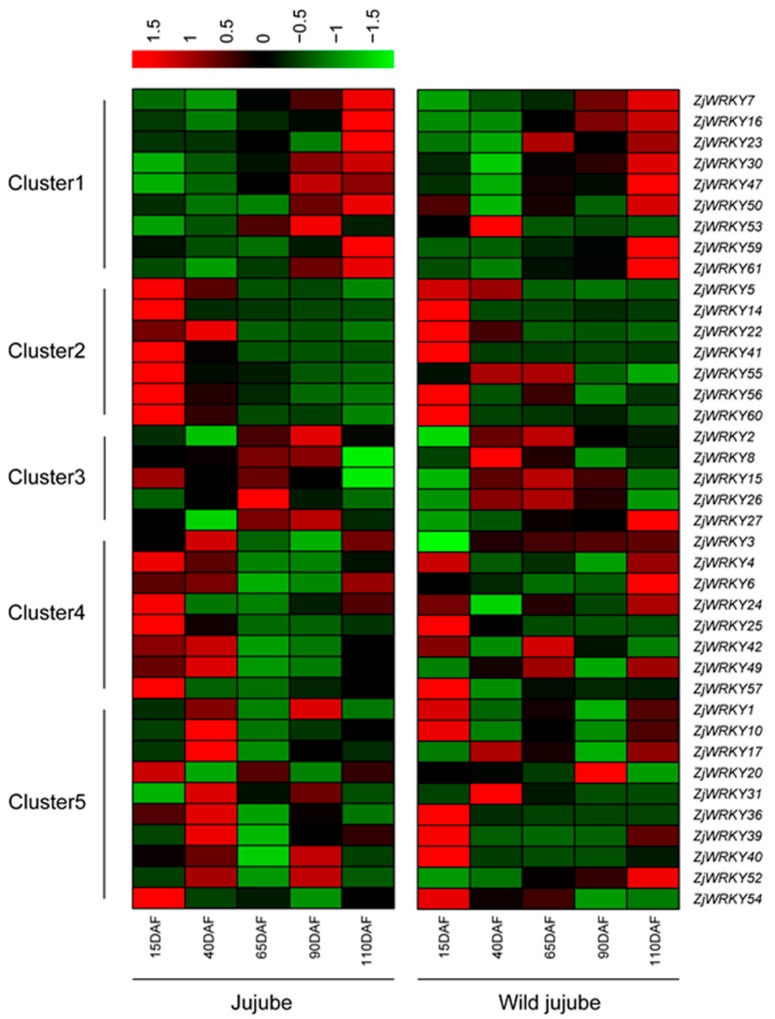
Transcriptional abundance of *ZjWRKY* genes during fruit ripening of cultivated jujube (‘Junzao’) and wild jujube (‘Qingjiansuanzao’). RPKM was used to measure the expression levels of the *ZjWRKY* genes. Red denotes high expression levels, and green denotes low expression levels. Clustering was done according to the expression level of *ZjWRKY* genes in jujube fruit.

Wild jujube is the wild type of jujube and genetically close to jujube, so their WRKY TFs might be similar with high possibility. On the other hand, wild jujube fruit is smaller and more acidic than jujube [24]. In order to identify *ZjWRKY* genes that might involve in fruit enlargement and sugar accumulation, we detected the expression level of *ZjWRKY* genes during the fruits development of wild and cultivated jujube. Based on the transcriptomic analysis, in total, there were 39 and 44 *ZjWRKY* genes with RPKM value ≥ 1 during fruit development and ripening of ‘Junzao’ jujube and ‘Qingjiansuanzao’ wild jujube, respectively. These 39 genes expressed in ‘Junzao’ fruit were divided into 5 clusters based on their expression level (Figure 7). In cluster 1 (9 genes), the expression level increased gradually. In cluster 2 (7 genes), the expression level decreased gradually. In cluster 3 (5 genes), the expression level increased first and then decreased, whereas in cluster 4 (8 genes), the expression level decreased first and then increased. In cluster 5 (10 genes), expression level was variable.

We further compared the transcript abundance of ZjWRKY genes between ‘Junzao’ and ‘Qingjiansuanzao’ during fruit ripening. We found that 20 of these 39 *ZjWRKY* genes showed similar expression patterns between the two accessions during the progress of fruit ripening. Additionally, the transcript abundance of 7 genes (*ZjWRKY5*, *24*, *25*, *30*, *41*, *57*, and *60*) in ‘Junzao’ fruit was always higher than that in ‘Qingjiansuanzao’ fruit, whereas the transcript abundance of 12 genes (*ZjWRKY1*, *2*, *3*, *6*, *14*, *15*, *16*, *26*, *49*, *52*, *54*, and *55*) in ‘Junzao’ fruit was always lower than those of ‘Qingjiansuanzao’. In addition, we found that *ZjWRKY* genes belonging to group I and group IId had a higher transcript abundance (RPKM ≥ 10) than those genes in the other groups.

### 3.7. Transcriptional Responses of ZjWRKY Genes to Salt, and Drought Stresses and ABA Treatment

To accurately evaluate the relative expression level of *ZjWRKY* genes by qRT-PCR analyses, we first established a standard curve for each gene to confirm the amplification efficiency of the primer sets. Among the 61 *ZjWRKY* genes, the transcription levels of 6 genes (*ZjWRKY9*, *18*, *22*, *43*, *45*, and *46*) were too low in all organs/tissues to establish standard curves.

**Figure 8:**
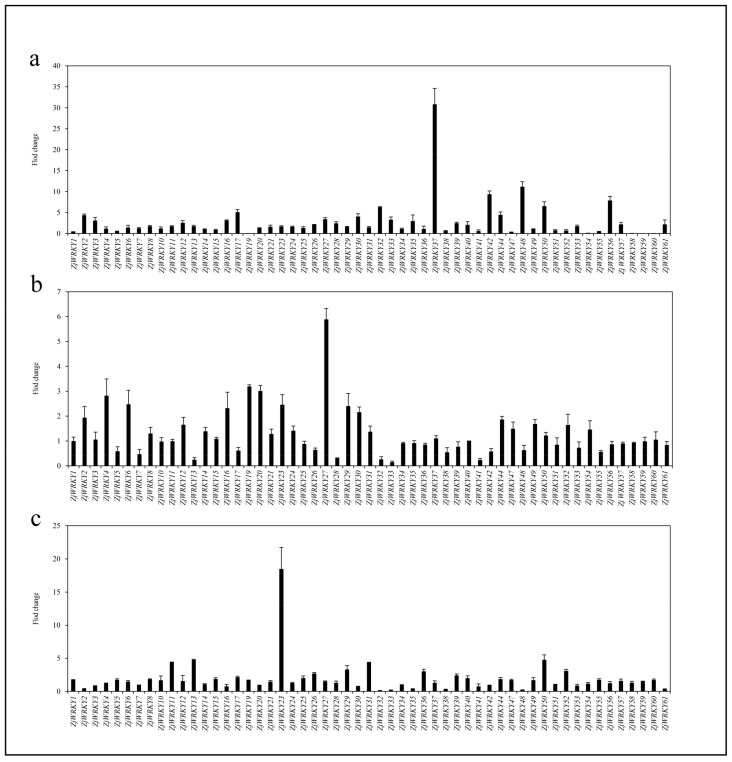
*ZjWRKY* genes expression in wild jujube seedlings under three different treatments. (**a**) Expression level of *ZjWRKY* genes under drought stress; (**b**) Expression level of *ZjWRKY* genes under NaCl stress; (**c**) Expression level of *ZjWRKY* genes under ABA treatment. The histograms represent the fold changes of expression level compared to the reference. The error bars represent standard deviation. *ZjWRKY* genes expression level were determined by real-time quantitative polymerase chain reaction (RT-qPCR) using *ZjUBQ* as a positive reference.

Under drought stress condition (10% water content), 21 *ZjWRKY* genes in the wild jujube seedlings were upregulated by over 2-fold, and two of them (*ZjWRKY37*, and *48*) increased >10-fold (Figure 8a). In particular, *ZjWRKY37* (group III) exhibited the highest expression level, with >30-fold upregulation. Analysis of *cis*-acting elements displayed that there were 2 ABA-responsive elements and 1 SA-responsive element in the promoter region of *ZjWRKY37* and there were 5 ABA-responsive elements and 2 MeJA-responsive elements in the promoter region of *ZjWRKY48*. In contrast, the expression levels of 9 *ZjWRKY* genes exhibited >2-fold decreases in these seedlings (Figure 8a).

Under salt stress condition (200 mM NaCl for 24 h), expression levels of 8 *ZjWRKY* genes showed >2-fold increases in the wild jujube seedlings. Among these genes, *ZjWRKY27* exhibited the highest expression level, with >5-fold increases. There were 3 MeJA-responsive elements, 1 SA-responsive element and 1 drought-responsive element in the promoter region of *ZjWRKY27*. On the other hand, 5 *ZjWRKY* genes exhibited >2-fold downregulation in these seedlings (Figure 8b).

For seedlings treated with 100 μM ABA for 24 h, the expression of 10 *ZjWRKY* genes were >2-fold upregulated and *ZjWRKY23* was mostly upregulated (group I), with >18-fold increases in the seedlings. In the *ZjWRKY23* promoter region, we identified 3 ABA-responsive elements, 1 SA-responsive element and 1 drought-responsive element. On the other hand, 8 *ZjWRKY* genes were downregulated by >2-fold in these seedlings (Figure 8c).

**Figure 9:**
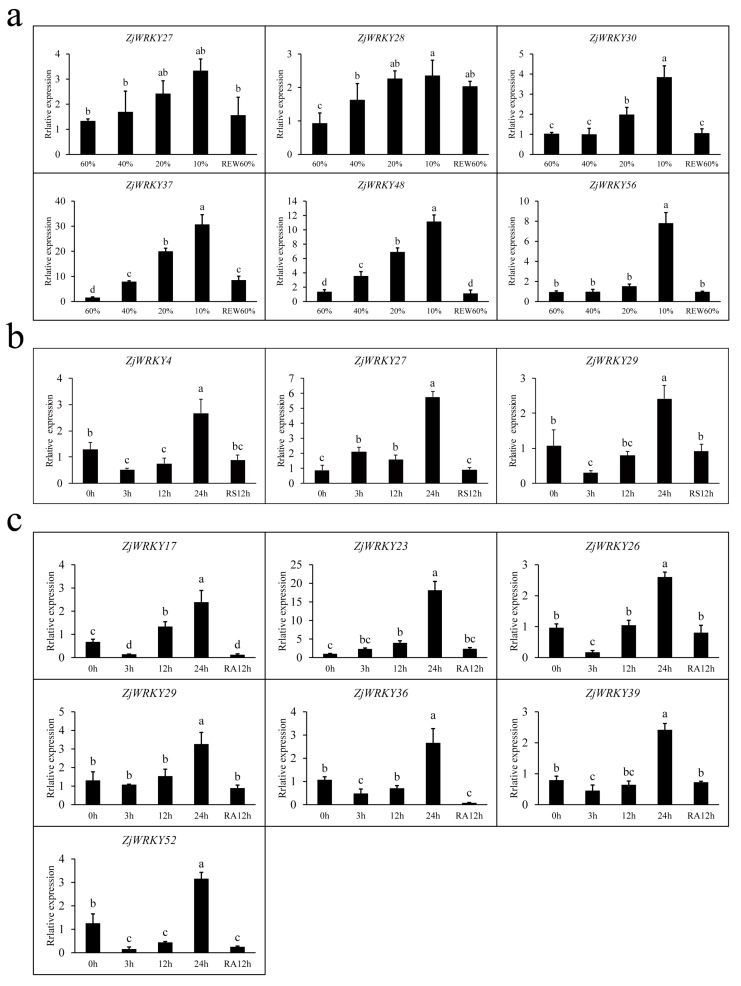
Expression profile of the *ZjWRKY* genes under three treatments. (**a**) Expression level of 6 *ZjWRKY* genes in soil moisture content 60%, 40%, 20%, 10%, and 60% after re-watering (REW60%); (**b**) Expression level of 3 *ZjWRKY* genes under 0 h, 3 h, 12 h, and 24 h NaCl treatment, and 12 h removal of NaCl (RS 12 h); (**c**) Expression level of 7 *ZjWRKY* genes after 0 h, 3 h, 12 h, and 24 h of ABA treatment, and 12 h after removal of ABA (RA 12 h). The histograms represent the relative expression level of *ZjWRKY* genes compared to the reference. Error bars represent standard deviations. The data were calculated using the method of 2^-ΔΔCt^. LSD detection was used to label different letters when there was a significant difference in expression (*p* < 0.05; *n* = 3).

We selected some genes which were regulated by >2-fold at transcript level after drought, salt and ABA treatment for further analysis. Under drought stress, as shown in Figure S7a, the maximum value of expression of different *ZjWRKY* genes appeared in different degrees of drought stress (Figure S8). It is worth noting that the expression levels of 5 genes (*ZjWRKY27*, *28*, *30*, *37*, *48*, and *56*) were negatively correlated with soil water content, that is, the expression level of these genes increased when the soil moisture reduced, but the expression level fell after rehydration (Figure 9a).

Among the selected 14 *ZjWRKY* genes (Figure S7b), there were 2, 1, 0, and 8 genes reaching their highest transcript level when treated with NaCl for 0, 3, 12, and 24 h, respectively. The transcription level of *ZjWRKY27* increased with the time of NaCl treatment and fell back after removal of NaCl. This pattern was similar with that under drought treatment (Figure 9b). *ZjWRKY4*, and *29* firstly decreased to their lowest level after 1 to 3 h NaCl, and then increased with the time of NaCl treatment, and fell back after removal of NaCl.

Under ABA treatment, the expression level of *ZjWRKY23* was positively correlated with the duration of ABA treatment. There were 6 *ZjWRKY* genes (*ZjWRKY17*, *26*, *29*, *36*, *39*, and *52*) that showed similar trends, except their transcription levels decreased during the first 3 h of ABA treatment. Interestingly, *ZjWRKY29* showed a similar trend under NaCl and ABA treatments (Figure 9c).

**Figure 10:**
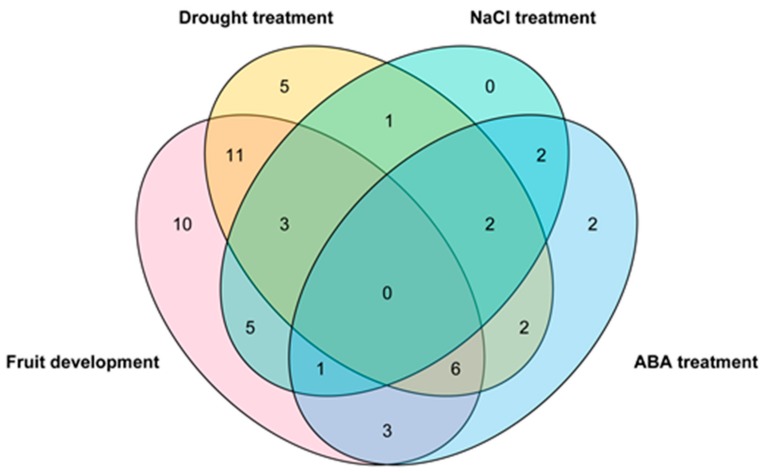
The Venn diagram of *ZjWRKY* genes related to fruit development, drought, NaCl, and ABA treatment.

Taken together, we constructed a Venn diagram of *ZjWRKY* genes involved in fruit development and response to drought, NaCl and ABA treatment. As showed in Figure 10, there were 39 *ZjWRKY* genes expressed during ‘Junzao’ jujube fruit development, and 30, 12, 18 *ZjWRKY* genes responded to drought, NaCl, and ABA treatments, respectively. Some *ZjWRKY* genes were involved in several processes. For example, 29 *ZjWRKY* genes expressed during the fruit development were also responded to abiotic stress. Among which, expression levels of *ZjWRKY32* and *33* in wild jujube seedlings changed when exposed to drought, NaCl and ABA treatments (Table S6). However, no genes were expressed in all events.

## 4. Discussion

### 4.1. Identification of the WRKY Family Members in Chinese Jujube

Accompanying the increasing number of plant genomes which were resolved, *WRKY* gene family, an important transcription factors family, has been identified in more and more plant species. In this study, we identified a total of 61 and 52 *WRKY* TFs from the ‘Junzao’ and ‘Dongzao’ jujube genome, respectively. Although most *WRKY* genes from ‘Dongzao’ have their corresponding members in ‘Junzao’ genome, there were some members that did not share between two accessions (Table S1). This difference also indicated more alleles existed among different cultivars. Of course, the difference in the number of WRKY TFs between ‘Junzao’ and ‘Dongzao’ might be also attributed to the difference in cultivar or sequencing strategy.

In contrast, there were 127 WRKY TFs in *M. domestica* belonging to Rosaceae that is neighbor with Rhamnaceae, about twice as large as in *Z. jujuba* [14]. This difference was contributed by the recent whole genome duplication event in the apple genome while no such event occurred in jujube genome [24].

### 4.2. ZjWRKY Expression Profile in Different Tissues

We analyzed the expression pattern of the *ZjWRKY* genes of ‘Junzao’ jujube in six different tissues. The results demonstrated variation in the expression patterns of *ZjWRKY* genes. In total, 7 genes were highly expressed in all jujube tissues. Highly expressed *WRKY* genes usually play important roles in plant development [23]. Therefore, we concluded that these 7 highly expressed *ZjWRKY* genes might be important regulatory factors in jujube development, although further studies are required to verify the function of these genes. In contrast, 2 *ZjWRKY* genes were expressed at low levels in all jujube tissues and 6 *ZjWRKY* genes were specifically expressed in only one tissue. Some low expression genes were induced by the specific environment. For example, *ZjWRKY46* was not detected in normal growing plants, but it was detected in seedlings treated with drought, NaCl, and ABA. However, *ZjWRKY18* cannot be detected in all samples. It might be activated by other conditions. Genes expressed at high levels in special organs usually play key roles in plant development [38]. In *A. thaliana*, *AtWRKY34* and *AtWRKY2* have higher expression levels in pollen and pollen tubes compared to other tissues. They were required for the development of male gametophytes [19]. In jujube, *ZjWRKY32*, and *33* were specifically expressed in roots. Their homologous gene, *AtWRKY72*, has a higher expression level in the lateral root cap and epidermis compared to other tissues [39]. *AtWRKY72* contributes to basal immunity in *A. thaliana* [40]. *ZjWRKY43* and *ZjWRKY45* were expressed only in flowers, *ZjWRKY48* was specifically expressed in stems, and *ZjWRKY41* was specifically expressed in young fruit. They might have specific functions in those corresponding tissues.

### 4.3. ZjWRKY Genes Associated with Jujube Fruit Development and Ripening

Fruit development and ripening is a complex process that includes cell division and expansion, the accumulation of sugar, fruit coloration, and other physiological processes. Studies have shown that a variety of TFs were involved in fruit development and ripening [41,42]. In pepper, *CaWRKY1* expression was strongly up-regulated in red fruit and may play an important role in fruit maturation [43]. In banana, the protein MaHIS1 and MaWRKY1 could interact with and regulate physiological processes like fruit ripening and stress responses [44]. In this study, we found 39 *ZjWRKY* genes expressed during jujube fruit development and ripening. The genes of groups I and IId had higher expression levels than those of other groups, which indicated *ZjWRKY* genes belonging to group I and IId play more important roles than other groups in jujube fruit development. Similar results as described above were also observed during the development of pepper fruit [23]. In this study, expression levels of 7 *ZjWRKY* genes were gradually decreased during the process of fruit ripening. We found the *cis*-acting regulatory element that responds to gibberellin signal from the promoter region of these genes. Moreover, the gibberellin content gradually decreased during the ripening of the ‘Dongzao’ jujube fruit [45]. Therefore, we speculate that these genes may be related to gibberellin.

Considering the huge change in fruit size and fruit taste between jujube and wild jujube, WRKY TFs might play a role in the difference of fruit ripening. By comparing *ZjWRKY* member expression patterns between jujube and wild jujube, we found that 7 *ZjWRKY* genes display higher transcript levels in jujube fruit than in wild jujube fruit while 12 *ZjWRKY* genes showed higher transcript levels in wild jujube fruit. Therefore, these *ZjWRKY* might control genes involved in fruit enlargement and quality development.

### 4.4. ZjWRKY Genes Involved in Response to Abiotic Stresses

WRKY TFs are widely involved in the regulation of plant responses to stress. In *A. thaliana*, *M. domestica* and *Sesamum indicum*, 18, 34, and 26 *WRKY* genes were associated with salt, waterlogging and drought stresses, respectively [14,34,46]. In this study, we found that 30, 14, and 18 *ZjWRKY* genes responded to drought stress, salt stress and ABA treatment, respectively. WRKY TFs could regulate plant responses to abiotic stress through ABA-dependent pathways. In *A. thaliana*, *AtWRKY46* modulates the development of *A. thaliana* lateral roots in osmotic/salt stress conditions via regulation of ABA signaling [47]. In this study, *ZjWRKY29* had a similar expression pattern with salt and ABA treatment, so this gene may respond to salt stress through ABA-dependent pathway. In addition, we found that *ZjWRKY27* had similar expression patterns under drought stress and salt stress (Figure 9a,b), indicates that it can function in jujube under different stresses. Previous studies have demonstrated that single transcription factor may function in several seemingly disparate signaling pathways. For example, *AtWRKY33* reacted to both heat treatment and salt treatment [48].

There were 5 genes (*ZjWRKY27*, *28*, *30*, *37*, *48*, and *56*) responding positively to drought stress. Among these 5 genes, *ZjWRKY48* is the orthology gene of *AtWRKY40*. In *A. thaliana*, ABA induction of *AtWRKY18* and *AtWRKY40* leads to increase in WRKY18 and WRKY40 proteins that form heterocomplexes through physical interactions. WRKY18/WRKY40 heterocomplex could activate *AtWRKY60* expression. *AtWRKY60* positively regulate plant responses to ABA [49]. Thus, some of them may play an important role in jujube response to drought stress.

Upon ABA treatment, the expression level of *ZjWRKY23* increased with ABA treatment. *AtWRKY33* is an orthologs of *ZjWRKY23*, which could upregulated in *A. thaliana* by ABA treatment [50]. Overexpression of *AtWRKY33* can increase NaCl tolerance in *A. thaliana* [51]. In addition, the expression level of *ZjWRKY27* increased with the duration of NaCl treatment. How those *ZjWRKY* genes regulating jujube responding to ABA needs be further elucidated by gene functional analyses.

In addition, according to the distribution pattern of *cis*-regulation elements in the promoter regions of ZjWRKY TFs, we found the distribution of cis-regulatory elements in the promotor regions of different *ZjWRKY TFs* was unnecessarily related with the gene expression pattern. Thus, their potential roles in gene regulation need to be validated in future research.

## 5. Conclusions

In this study, we identified and compared 61 and 52 ZjWRKY TFs from two famous jujube cultivars, i.e., ‘Junzao’ and ‘Dongzao’, respectively. We characterized their distribution on chromosomes, conserved motifs, gene structure, *cis*-acting regulatory elements in promoter regions, and evolutionary relationships. This difference of ‘Junzao’ and ‘Dongzao’ ZjWRKY TFs might be attributed to the difference in sequencing strategies or genetic background of two cultivars. Furthermore, we found *ZjWRKY* genes of ‘Junzao’ showed different expression pattern in the different tissues. Among the 61 genes, 7 *ZjWRKY* genes highly expressed in various tissues. Transcriptome analysis showed that 39 *ZjWRKY* genes expressed during ‘Junzao’ fruit ripening, among which 19 *ZjWRKY* genes were differentially expressed between ‘Junzao’ and ‘Qingjiansuanzao’, indicating their possible roles regulating sugar accumulation and acid metabolism. On the other hand, expression levels of 30, 14, and 18 *ZjWRKY* genes changed when wild jujube seedlings were exposed to drought, NaCl and ABA treatments, respectively. Among these genes, 5 and 1 *ZjWRKY* genes were strongly correlated with the degree of drought and salt stress, respectively. Taken together, our study revealed the functional diversity of WRKY TFs and provided candidate *WRKY* genes for future breeding of drought/salt-tolerance jujube trees.

## Figures and Tables

**Table 1:** Identified *WRKY* genes in the genome of *Ziziphus jujuba* ‘Junzao’.

Name Symbol	Chromosome	Peptide Length	pI	MW	CDS Number	Group	Conserved Heptapeptide	Zinc-Finger Type
*ZjWRKY1*	Chr1	329	9.57	35	3	II d	WRKYGQK	CX5CX23HXH
*ZjWRKY2*	Chr1	286	5.62	31	3	II e	WRKYGQK	CX5CX23HXH
*ZjWRKY3*	Chr1	337	6.37	37	3	II c	WRKYGQK	CX4CX23HXH
*ZjWRKY4*	Chr1	759	5.57	82	5	I	WRKYGQK WRKYGQK	CX4CX22HXH CX4CX23HXH
*ZjWRKY5*	Chr1	490	5.70	52	3	II e	WRKYGQK	CX5CX23HXH
*ZjWRKY6*	Chr1	634	6.12	68	6	II b	WRKYGQK	CX5CX23HXH
*ZjWRKY7*	Chr1	522	5.12	57	2	I	WRKYGQK WRKYGQK	CX4CX22HXH CX4CX23HXH
*ZjWRKY8*	Chr1	591	6.12	65	7	I	WRKYGQK WRKYGQK	CX4CX22HXH CX4CX23HXH
*ZjWRKY9*	Chr1	536	6.26	58	6	II b	WRKYGQK	CX5CX19HXH
*ZjWRKY10*	Chr1	317	8.68	35	5	II a	WRKYGQK	CX5CX23HXH
*ZjWRKY11*	Chr2	648	6.74	70	6	II b	WRKYGQK	CX5CX23HXH
*ZjWRKY12*	Chr2	200	9.21	23	2	II c	WRKYGQK	CX4CX23HXH
*ZjWRKY13*	Chr2	648	6.74	70	6	II b	WRKYGQK	CX5CX23HXH
*ZjWRKY14*	Chr3	346	6.76	39	3	II c	WRKYGQK	CX4CX23HXH
*ZjWRKY15*	Chr3	393	8.60	43	3	I	WRKYGQK WRKYGQK	CX4CX22HXH CX4CX23HXH
*ZjWRKY16*	Chr3	619	6.21	67	5	II b	WRKYGQK	CX5CX23HXH
*ZjWRKY17*	Chr3	283	5.46	31	3	II e	WRKYGQK	CX5CX23HXH
*ZjWRKY18*	Chr3	161	8.79	18	2	III	WRKYGQK	N
*ZjWRKY19*	Chr3	514	7.63	56	4	II b	WRKYGQK	CX5CX23HXH
*ZjWRKY20*	Chr3	502	6.52	55	4	I	WRKYGQK WRKYGQK	CX4CX22HXH CX4CX23HXH
*ZjWRKY21*	Chr5	398	7.08	44	4	II c	WRKYGQK	CX4CX23HXH
*ZjWRKY22*	Chr5	560	5.81	61	5	I	WRKYGQK WRKYGQK	CX4CX22HXH CX4CX23HXH
*ZjWRKY23*	Chr5	593	7.21	65	5	I	WRKYGQK WRKYGQK	CX4CX22HXH CX4CX23HXH
*ZjWRKY24*	Chr6	362	9.65	41	3	II d	WRKYGQK	CX5CX23HXH
*ZjWRKY25*	Chr6	336	6.43	37	3	II c	WRKYGQK	CX4CX23HXH
*ZjWRKY26*	Chr6	558	7.22	61	6	II b	WRKYGQK	CX5CX23HXH
*ZjWRKY27*	Chr6	290	5.27	32	3	II e	WRKYGQK	CX5CX23HXH
*ZjWRKY28*	Chr6	196	9.34	22	2	II c	WRKYGQK	CX4CX23HXH
*ZjWRKY29*	Chr6	504	5.50	56	5	II b	WRKYGQK	CX5CX23HXH
*ZjWRKY30*	Chr7	415	6.99	46	4	I	WRKYGQK WRKYGQK	CX4CX22HXH CX4CX23HXH
*ZjWRKY31*	Chr7	311	5.65	34	3	II c	WRKYGQK	CX4CX23HXH
*ZjWRKY32*	Chr7	636	6.62	69	5	II b	WRKYGQK	CX5CX23HXH
*ZjWRKY33*	Chr7	632	6.45	68	5	II b	WRKYGQK	CX5CX23HXH
*ZjWRKY34*	Chr8	474	5.19	52	3	II e	WRKYGQK	CX5CX23HXH
*ZjWRKY35*	Chr8	392	5.90	44	3	III	WRKYGQK	CX7CX23HXC
*ZjWRKY36*	Chr8	345	5.39	39	3	III	WRKYGQK	CX7CX23HXC
*ZjWRKY37*	Chr8	405	6.64	45	3	III	WRKYGQK	CX7CX23HXC
*ZjWRKY38*	Chr9	393	4.93	45	2	II e	WRKYGQK	CX5CX23HXH
*ZjWRKY39*	Chr9	543	7.09	60	5	I	WRKYGQK WRKYGQK	CX4CX22HXH CX4CX23HXH
*ZjWRKY40*	Chr9	371	5.26	42	3	III	WRKYGQK	CX7CX23HXC
*ZjWRKY41*	Chr9	300	5.35	33	3	II c	WRKYGQK	CX4CX23HXH
*ZjWRKY42*	Chr9	320	5.25	36	3	III	WRKYGQK	CX7CX23HXC
*ZjWRKY43*	Chr9	318	8.08	36	3	III	WRKYGQK	CX7CX23HXC
*ZjWRKY44*	Chr9	308	5.84	35	3	III	WRKYGQK	CX7CX23HXC
*ZjWRKY45*	Chr9	304	5.25	34	3	III	WRQYGQK	CX7CX23HXC
*ZjWRKY46*	Chr9	316	6.46	36	3	III	WRKYGQK	CX7CX23HXC
*ZjWRKY47*	Chr10	367	9.64	39	3	II d	WRKYGQK	CX5CX23HXH
*ZjWRKY48*	Chr10	214	9.32	23	3	II a	WRKYGQK	CX5CX23HXH
*ZjWRKY49*	Chr10	372	9.57	40	3	II d	WRKYGQK	CX5CX23HXH
*ZjWRKY50*	Chr10	320	8.65	35	4	II a	WRKYGQK	CX5CX23HXH
*ZjWRKY51*	Chr11	227	9.11	25	3	II c	WRKYGQK	CX4CX23HXH
*ZjWRKY52*	Chr11	374	5.16	41	3	II c	WRKYGQK	CX4CX23HXH
*ZjWRKY53*	Chr11	224	5.37	25	3	II e	WRKYGQK	CX5CX23NXH
*ZjWRKY54*	Chr11	356	5.92	39	3	II e	WRKYGQK	CX5CX23HXH
*ZjWRKY55*	Chr11	478	9.09	52	5	I	WRKYGQK WRKYGQK	CX4CX22HXH CX4CX23HXH
*ZjWRKY56*	Chr12	243	8.66	28	3	II c	WRKYGQK	CX4CX23HXH
*ZjWRKY57*	Chr12	196	6.73	22	3	II c	WRKYGKK	CX4CX23HXH
*ZjWRKY58*	Chr12	355	5.23	41	3	III	WRKYGQK	CX7CX23HXC
*ZjWRKY59*	Chr12	329	9.53	36	3	II d	WRKYGQK	CX5CX23HXH
*ZjWRKY60*	Conting12.1	159	5.46	18	3	II c	WRKYGKK	CX4CX23HXH
*ZjWRKY61*	Conting111	237	7.65	26	4	II c	WRKYGQK	CX4CX23HXH

CDS number: the number of coding sequences.

## Supplementary Materials

The following are available online at https://www.mdpi.com/2073-4425/10/5/360/s1. Figure S1: The *WRKY* genes identification, evolutionary relationships, motif characteristics, genes structure, and *cis*-acting element types and numbers of WRKY TFs from ‘Dongzao’ jujube. Figure S2: Phylogenetic analysis of the WRKY proteins from Chinese jujube, *Arabidopsis thaliana* and *Solanum lycopersicum*. Multiple sequence alignments of WRKY amino acid sequences were performed using ClustalW. The phylogenetic tree was constructed using MEGA7.0 with the Maximum Likelihood method. Figure S3: Multiple sequence alignment of ‘Junzao’ WRKY conserved domains. Dashed lines indicate the gap. The overall height of each stack on the logo illustrates the sequence conservation at each position. Each residue letter height symbolizes the relative frequency of the corresponding residue. Figure S4: Detailed information of the ‘Junzao’ and ‘Dongzao’ motif obtained by Multiple Em for Motif Elicitation (MEME). Figure S5: Expression levels of *ZjWRKY18*, and *46* after drought, NaCl, ABA treatments. Figure S6: Expression level of ZjWRKY43, and 45 in sepals, disks, pistils, and stamen. Figure S7: Expression profile of the *ZjWRKY* genes under three treatments. a: Expression level of 30 *ZjWRKY* genes in soil moisture content 60%, 40%, 20%, 10%, and 60% after re-watering (REW60%); b: Expression level of 14 *ZjWRKY* genes under 0 h, 3 h, 12 h, and 24 h NaCl treatment, and 12 h removal of NaCl (RS 12 h); c: Expression level of 18 *ZjWRKY* genes after 0 h, 3 h, 12 h, and 24 h of ABA treatment, and 12 h after removal of ABA (RA 12 h). The histograms represent the relative expression level of *ZjWRKY* genes compared to the reference. Error bars represent standard deviations. The data were calculated using the method of 2^-ΔΔCt^. LSD detection was used to label different letters when there was a significant difference in expression (*p* < 0.05; n = 3). Figure S8: Growth status of wild jujube seedlings under drought stress. Table S1: Comparison of ZjWRKY protein sequences of ‘Dongzao’ and ‘Junzao’ using BLAST-2.3.31+. Table S2: Identified WRKY genes in the genome of Ziziphus jujuba ‘Dongzao’. Table S3: Identified cis-acting regulatory elements in the putative promoter regions of ‘Junzao’ WRKY genes. Table S4: Designed primer sets used in this study. Table S5: Relative expression levels of the ZjWRKY genes in six tissues/organs of ’Junzao’ jujube using Image J. Table S6: ZjWRKY genes related to fruit development, drought, NaCl and ABA treatment.
